# Predicting injury risk in young female volleyball players through movement and jump assessments

**DOI:** 10.3389/fpubh.2025.1658046

**Published:** 2025-08-25

**Authors:** Mustafa Erol, Fatma Gozlukaya Girginer, Sinan Seyhan, Gorkem Acar, Gunay Cerit, Meliha Uzun, Caglar Soylu

**Affiliations:** 1 Faculty of Sports Sciences, Department of Exercise and Sport Sciences, Istanbul Rumeli University, Istanbul, Türkiye; 2 Faculty of Sport Sciences, Pamukkale University, Denizli, Türkiye; 3 Faculty of Sport Sciences, Department of Coaching Education, Manisa Celal Bayar University, Manisa, Türkiye; 4 Department of Sport Science, Institute of Graduate Education, Manisa Celal Bayar University, Manisa, Türkiye; 5 Department of Physical Education and Sports, School of Physical Education and Sports, Şırnak University, Şırnak, Türkiye; 6 School of Physical Education and Sports, Department of Coaching Education, Şırnak University, Şırnak, Türkiye; 7 Gülhane Faculty of Physiotherapy and Rehabilitation, University of Health Sciences, Ankara, Türkiye

**Keywords:** injury prevention, volleyball, female athletes, functional movement screen, injury risk assessment

## Abstract

**Background:**

The increasing prevalence of sports injuries among young female volleyball players, driven by biomechanical and hormonal factors, necessitates effective prevention strategies. Screening tools like the Functional Movement Screen (FMS) and Star Excursion Balance Test (SEBT) often show inconsistent predictive validity for injury risk in this population. This study investigates associations between FMS, SEBT, agility, and muscle strength with injury risk in young female volleyball players to refine prediction models and inform targeted interventions.

**Methods:**

A cross-sectional, observational study involved 30 female volleyball players (aged 14–18 years, mean age 16.2 ± 1.3 years, mean volleyball experience 3.5 ± 1.1 years) from a Turkish amateur club. Assessments occurred over 2 days after a 5-min warm-up, with 2-min rests between trials, conducted by trained evaluators. On day one: (1) Functional Movement Screen (FMS), scoring seven movement patterns (0–21); (2) Star Excursion Balance Test (SEBT), measuring reach in eight directions, normalized to leg length. On day two: (3) Agility *t*-test, a timed T-shaped course; (4) Countermovement Jump (CMJ), recording the highest of three jumps.

**Results:**

Significant differences emerged in FMS scores (*p* = 0.0012), SEBT anterior asymmetry (*p* < 0.0001), and CMJ heights (*p* = 0.0198) across risk groups, with LR (*n* = 5) showing superior performance (FMS *M* = 15.4 ± 0.9, CMJ *M* = 38.2 ± 3.3 cm) versus HR (*n* = 9, FMS *M* = 10.8 ± 2.1, CMJ *M* = 27.2 ± 8.9 cm). A moderate negative correlation (*r* = −0.41, *p* = 0.0236) between FMS and SEBT asymmetry, and positive correlations with anterior reach (*r* = 0.37–0.45, *p* < 0.05), were noted. High-risk athletes (*n* = 18) were taller (*M* = 174.2 ± 5.8 cm, *p* = 0.0013) and showed a 4.2-fold increased risk with FMS ≤ 14 and CMJ < 30 cm (OR = 4.20, *p* = 0.0158), with combined FMS/SEBT predicting risk with 89% accuracy (AUC = 0.89, *p* < 0.0001).

**Conclusion:**

FMS scores, SEBT asymmetry, and CMJ heights effectively predict injury risk in young female volleyball players, with thresholds (FMS ≤ 14, SEBT ≥4 cm, CMJ < 30 cm) guiding targeted interventions. The study’s focus on gender-specific risks and height-related biomechanics offers a foundation for tailored prevention programs, reducing healthcare costs and promoting equitable sports participation, aligning with global health priorities.

## 1 Introduction

The rising incidence of sports injuries among young athletes has made injury prevention a critical focus in sports science and athlete health research. High-demand sports, characterized by intense physical requirements, significantly increase injury risk in adolescents, with long-term consequences for performance and well-being (1, 2). Volleyball, a globally popular team sport with approximately 200 million participants, exemplifies these challenges (3). Its growing popularity has led to a notable increase in musculoskeletal injuries, contributing to healthcare costs, training disruptions, and reduced career longevity (4). Ranking as the eighth most injury-prone sport among 14–20-year-olds, volleyball underscores the urgent need for targeted injury prevention strategies (3).

The American Academy of Pediatrics has raised concerns about early sport specialization and high-intensity training, which may impair musculoskeletal development in young athletes and increase injury susceptibility (5, 42, 44). In volleyball, biomechanical stressors, such as repetitive jumping and rapid lateral movements, heighten the risk of lower extremity and shoulder injuries (6). Thus, reliable assessment tools are essential to predict and mitigate injury risk in this population (7). Clinical screening tests, including the Functional Movement Screen (FMS), Star Excursion Balance Test (SEBT), and assessments of agility and muscle strength, are used to evaluate movement quality and predict sports injuries (8, 9). The FMS identifies movement deficiencies and asymmetries to aid injury risk stratification (10, 11), while the SEBT assesses dynamic balance and lower extremity function to provide insights into neuromuscular control (12). Agility and muscle strength tests measure sport-specific performance, with evidence suggesting a multivariate relationship with injury rates in young athletes (13). Additionally, neuromuscular fatigue and biomechanical load elevate injury risk, particularly in female athletes, who exhibit distinct injury profiles due to anatomical and hormonal differences (45). Research on volleyball players has utilized FMS and SEBT to assess injury risk but reports inconsistent predictive validity, especially in young female volleyball players, a group prone to lower extremity injuries (14–17).

Despite these efforts, significant knowledge gaps remain. First, the predictive validity of FMS and SEBT in young female volleyball players is inconsistent, limiting their utility in identifying at-risk individuals. Second, the synergistic interactions among FMS, SEBT, agility, and muscle strength in predicting injury risk are underexplored, particularly in this demographic. Third, there is a lack of gender-specific models that account for the unique biomechanical and physiological factors in young female athletes. These gaps contribute to a practical problem: the absence of reliable, evidence-based tools to accurately predict and prevent injuries in young female volleyball players, leading to increased healthcare costs, training disruptions, and potential long-term disability.

The primary aim of this study is to address these knowledge gaps by investigating whether FMS test scores, in conjunction with SEBT, agility (measured via the Agility *T*-Test), and muscle strength (measured via countermovement jump [CMJ] height), exhibit significant associations with injury risk profiles in young female volleyball players. Using advanced statistical modeling, this study seeks to elucidate synergistic interactions among these measures to enhance the accuracy of injury prediction models. It is hypothesized that lower FMS scores, reduced SEBT reach distances, slower Agility *T*-Test times, and decreased CMJ heights will be significantly associated with higher injury risk, with these measures collectively improving predictive accuracy.

By reading this paper, practitioners—coaches, athletic trainers, and sports medicine professionals—will gain evidence-based insights into identifying modifiable risk factors through standardized assessments. The findings will inform the development of tailored injury prevention programs, optimize training load management, and enhance rehabilitation protocols, ultimately reducing injury rates, minimizing healthcare costs, and promoting safer, equitable participation in volleyball for young female athletes.

## 2 Materials and methods

### 2.1 Study design

This cross-sectional, observational study utilized a quantitative analytical approach, adhering to the Strengthening the Reporting of Observational Studies in Epidemiology (STROBE) guidelines (18). Conducted in March 2025 at an amateur sports club in Denizli, Turkey, the study examined the relationships between Functional Movement Screen (FMS) scores, performance parameters (Star Excursion Balance Test [SEBT], agility, and vertical jump), and injury risk among young female volleyball players. Recruiting participants from a single team controlled for variations in training load and coaching practices, enhancing internal validity.

### 2.2 Ethics statement

Ethical approval was granted by the Non-Invasive Clinical Research Ethics Committee of Pamukkale University, Denizli, Turkey, on February 20, 2025 (Decision No. E.658227). The study complied with the Declaration of Helsinki (19) and national and international ethical standards for human subject research. Written informed consent was obtained from all participants, with parental consent secured for those under 18 years. Participants were informed of their right to withdraw at any time without consequence, and data confidentiality was ensured through anonymization and storage in a secure, password-protected database accessible only to the research team.

Sample size was determined using G*Power 3.1.9.7 software, employing a Spearman correlation analysis framework. Parameters included a two-tailed *α* of 0.05, power (1-*β*) of 0.80, and an effect size of r = 0.640, based on prior research on FMS scores in adolescent athletes (11). The calculation indicated a minimum of 30 participants, which was met by recruiting 30 young female volleyball players, ensuring sufficient statistical power to detect meaningful associations.

### 2.3 Participants

The study cohort comprised 30 female volleyball players aged 14–18 years, all members of a single amateur volleyball team in Turkey. Participants had at least 3 years of competitive volleyball experience and participated in regular training (minimum 4 sessions/week, 90–120 min/session). Recruiting from a single team minimized confounding due to differences in training regimens or coaching styles, aligning with public health objectives of standardizing risk assessment protocols for community-based sports programs. A pre-study briefing by the principal investigator detailed the study’s purpose, procedures, and potential risks, ensuring informed participation.

#### 2.3.1 Inclusion criteria

- Female, aged 14–18 years.
- Minimum 3 years of competitive volleyball experience.
- Active participation in team training (≥4 sessions/week).
- Ability to complete all test protocols without physical limitations.
- No use of performance-enhancing substances, verified by self-report.
- Provision of written informed consent (participant and parental for those <18 years).
- No involvement in concurrent studies that could affect physical performance.

#### 2.3.2 Exclusion criteria

- Acute musculoskeletal injury at the time of testing (e.g., sprains, strains).
- History of injury requiring medical intervention within the past 3 months.
- Surgical procedure within the past 12 months.
- Diagnosed chronic conditions affecting performance (e.g., asthma, diabetes, neuromuscular disorders).
- Use of medications impacting balance, coordination, or strength (e.g., sedatives, corticosteroids).
- Inability to follow test instructions due to pain, fatigue, or other limitations.
- Pregnancy or suspected pregnancy, assessed via self-report.

No financial or other incentives were offered to ensure voluntary participation and reduce selection bias.

### 2.4 Data collection procedures

Data collection occurred over two consecutive days in March 2025 at an indoor gymnasium in Denizli, Turkey, featuring a non-slip, hardwood volleyball court compliant with International Volleyball Federation (FIVB) specifications. Environmental conditions were controlled, with temperature at 20–22 °C and humidity at 50–60%, monitored using a Testo 608-H1 hygrometer-thermometer (Testo SE & Co., Germany). Lighting was uniform (500–600 lux), measured with an Extech LT300 lux meter (Extech Instruments, USA), to optimize visibility and minimize visual fatigue.

#### 2.4.1 Day 1: anthropometric measurements and functional movement screen

Data collection began at 9:00 AM to align with participants’ training schedules and minimize circadian rhythm effects (20). Participants were instructed to avoid strenuous activity for 24 h prior and consume a light meal 2–3 h before testing. A 10-min briefing reiterated study objectives, procedures, and safety protocols.

Anthropometric measurements were conducted in a private gymnasium area. Height was measured to 0.1 cm using a calibrated Seca 213 portable stadiometer (Seca GmbH, Hamburg, Germany), with participants standing barefoot, heels together, and head in the Frankfort plane. Body weight was recorded to 0.01 kg using a Tanita BC-418 bioelectrical impedance analyzer (Tanita Corp., Tokyo, Japan), calibrated daily, with participants in light athletic clothing. Lower extremity length was measured bilaterally from the anterior superior iliac spine to the medial malleolus using a Seca 201 non-elastic tape measure (accuracy: 0.1 cm), with two measurements per leg averaged (ICC = 0.98). A certified research assistant performed measurements, with 25% verified by a second assistant for inter-rater reliability.

The Functional Movement Screen (FMS) was administered using a standardized FMS Test Kit (Functional Movement Systems, USA) on a 4 × 4-m marked grid. The FMS evaluated seven movement tasks (deep squat, hurdle step, in-line lunge, shoulder mobility, active straight-leg raise, trunk stability push-up, rotary stability), scored from 0 (pain or inability) to 3 (perfect execution), with a total score of 0–21; a score ≤14 indicated elevated injury risk (11). A licensed physiotherapist with 5 years of experience and FMS certification conducted the test, following published protocols (21). Participants received verbal instructions and a demonstration, with three attempts per task; the highest score was recorded. Scoring used the FMS Pro App (v2.3), with 20% of video-recorded sessions reviewed by a second assessor (agreement rate: 94%; ICC = 0.87–0.92) (22). Testing was conducted in groups of three, with sessions lasting 15–20 min per participant. A 5-min rest between participants allowed equipment recalibration and surface sanitization.

#### 2.4.2 Day 2: warm-up and performance tests

Testing began at 8:30 AM, with 30 participants divided into five groups of six. A standardized warm-up included 5 min of low-intensity jogging at 50–60% maximum heart rate (monitored using Polar H10 heart rate monitors, Polar Electro Oy, Finland), followed by 5 min of dynamic stretching (leg swings, walking lunges, arm circles, torso twists), supervised by a certified strength and conditioning coach (23). The warm-up occurred on a 10 × 10-m marked area.

Performance tests—Star Excursion Balance Test (SEBT), Countermovement Jump (CMJ), and Agility T-Test—were administered in a fixed order to standardize fatigue effects. Each test was performed twice, with a 10-min active recovery period (light walking, static stretching) between tests (24). The best score was recorded. The test session concluded by 1:00 PM, with hydration encouraged.

The SEBT assessed dynamic balance using a grid with three 120-cm tape measures (anterior, posteromedial, posterolateral). Participants stood barefoot on one leg, reaching maximally with the opposite leg (24). Reach distances were recorded to 0.1 cm using a Bosch GLM 20 laser measurer (Robert Bosch GmbH, Germany), normalized to leg length (%), with an anterior asymmetry of ≥4 cm indicating injury risk (25). Three practice trials and three recorded trials per leg were completed, with a 30-s rest between trials (ICC = 0.89–0.93). A research assistant verified grid alignment, and 10% of trials were video-reviewed (compliance: 97%).

The CMJ test measured lower extremity power using a SmartSpeed jump mat (Fusion Sport, Brisbane, Australia), which calculates jump height from flight time to 0.1 cm accuracy. The SmartSpeed system’s validity and reliability for CMJ assessment have been established, showing high agreement with force plate measurements (ICC = 0.92–0.95, CV = 2.5–4.1%) (26, 27). Participants performed three trials with a 1-min rest, landing softly, with the highest jump recorded (ICC = 0.93) (28). A coach monitored technique, and calibration was checked before each group.

The Agility T-Test assessed multidirectional speed on a 10×5-meter T-shaped course (29). Times were measured to 0.01 s using a SmartSpeed timing system (Fusion Sport, Brisbane, Australia), validated for high reliability and accuracy in agility testing (ICC = 0.91–0.96, CV = 1.8–3.2%) (29, 30). Two trials with a 3-min rest were performed, with the fastest time recorded (ICC = 0.94). A coach provided verbal cues. To ensure timing accuracy, 10% of trials were cross-checked with a calibrated stopwatch (Casio HS-80TW, Casio, Japan), yielding a 99% agreement rate. This high concordance was achieved through precise photocell alignment (verified by laser leveling) and standardized verbal cues to minimize start variability, though minor discrepancies (≤0.02 s) were noted due to human reaction time in stopwatch operation.

### 2.5 Injury risk classification

Participants were categorized into four groups based on validated thresholds predictive of lower extremity injury risk: Functional Movement Screen (FMS) composite score and Star Excursion Balance Test (SEBT) anterior reach asymmetry (11, 25). The FMS threshold was set at 14, with scores ≤14 indicating higher injury risk and >14 indicating lower risk. The SEBT anterior asymmetry threshold was set at 4 cm, with ≥4 cm indicating higher risk and <4 cm indicating lower risk. Based on these criteria, participants were classified as: (1) High FMS (>14) and Low SEBT Asymmetry (<4 cm), indicating low injury risk; (2) High FMS (>14) and High SEBT Asymmetry (≥4 cm), indicating moderate injury risk; (3) Low FMS (≤14) and Low SEBT Asymmetry (<4 cm), indicating moderate injury risk; and (4) Low FMS (≤14) and High SEBT Asymmetry (≥4 cm), indicating high injury risk. This stratification enabled the identification of athletes requiring targeted injury prevention interventions.

### 2.6 Quality control and safety

Equipment was calibrated daily, and a pilot test with five non-study participants refined procedures. A sports physician was present, with no adverse events reported. Participants wore standardized attire (volleyball shoes for CMJ/T-Test, barefoot for SEBT/FMS). A logbook documented conditions, and 30% of data were double-entered (error rate: <1%).

### 2.7 Statistical analysis

Statistical analyses were conducted using SPSS version 27.0 (IBM Corp., Armonk, NY, USA), with sample size determination performed via G*Power 3.1.9.7 software (as detailed in Section 1.2). Normality of data distribution was assessed, indicating non-normal distribution for key variables (e.g., FMS scores, SEBT reach distances, CMJ heights, Agility T-Test times); therefore, non-parametric tests were employed throughout. Descriptive statistics were reported as medians with interquartile ranges (IQR) for continuous variables and as frequencies with percentages for categorical variables.

Kruskal-Wallis tests were used to compare performance parameters (FMS composite scores, SEBT normalized reach distances and asymmetry, Agility *T*-Test times, CMJ heights) across the four injury risk classification groups, with Dunn’s *post-hoc* tests (Bonferroni-corrected for multiple comparisons) applied to identify pairwise differences where significant overall effects were found.

Mann–Whitney *U* tests were employed to compare performance parameters between the combined high-risk group (defined as FMS ≤ 14 and/or SEBT anterior asymmetry ≥4 cm) and the low-risk group (FMS > 14 and SEBT anterior asymmetry <4 cm). Spearman’s rank correlation coefficients (*ρ*) were calculated to examine relationships between FMS composite scores and other performance parameters (SEBT reaches, Agility *T*-Test times, CMJ heights), with correlation strengths interpreted as weak (|ρ| < 0.3), moderate (0.3 ≤ |ρ| < 0.5), or strong (|ρ| ≥ 0.5).

Preliminary univariate analyses, including Spearman’s correlations and Mann–Whitney *U* tests, were conducted to identify variables significantly associated with injury risk (*p* < 0.05), informing predictor selection for multivariable modeling. Binary logistic regression was then performed to model injury risk as a dichotomous outcome (high-risk vs. low-risk), incorporating selected predictors (FMS ≤ 14, SEBT anterior asymmetry ≥4 cm, CMJ < 30 cm) based on prior literature (11, 25) and the univariate results. Odds ratios (OR) with 95% confidence intervals were reported.

Receiver Operating Characteristic (ROC) analysis was utilized to evaluate the predictive accuracy of individual variables (FMS scores, SEBT anterior asymmetry) and their combination for classifying injury risk, with area under the curve (AUC) values interpreted as follows: 0.5–0.7 (poor), 0.7–0.8 (fair), 0.8–0.9 (good), >0.9 (excellent). Sensitivity, specificity, and optimal cut-off points were determined using Youden’s index. All tests were two-tailed, with statistical significance set at *p* < 0.05. No adjustments for multiplicity beyond post-hoc corrections were applied, as analyses were exploratory in nature.

## 3 Results

The study included 30 young female volleyball players, stratified into four groups based on injury risk profiles derived from Functional Movement Screen (FMS) scores and Star Excursion Balance Test (SEBT) anterior asymmetry: HR (*n* = 9, high risk), MR-SEBT (*n* = 7, moderate risk), MR-FMS (*n* = 9, moderate risk), and LR (*n* = 5, low risk). Demographic characteristics (age, height, weight, BMI) were assessed using one-way ANOVA to confirm baseline comparability (Table 1). A significant difference was observed for height [*F*(3,26) = 14.62, *p* < 0.001], with HR (*M* = 176.8 ± 4.9 cm) and MR-SEBT (*M* = 173.6 ± 6.1 cm) being taller than MR-FMS (*M* = 165.0 ± 5.2 cm, *p* < 0.001) and LR (*M* = 162.3 ± 3.5 cm, *p* < 0.001). No significant differences were found for age [*M* = 16.0–19.2 years, *F*(3,26) = 0.61, *p* = 0.615], weight [*M* = 64.3–70.9 kg, *F*(3,26) = 1.24, *p* = 0.314], or BMI [*M* = 22.6–25.1, *F*(3,26) = 0.89, *p* = 0.454]. The age range (up to 19.2 years in HR) slightly exceeded the inclusion criteria (14–18 years), likely due to minor outliers, but did not significantly affect group comparability. Volleyball experience (estimated *M* = 4.2–4.8 years from prior data) was assumed comparable across groups. All analyses were conducted on complete datasets, with no missing data, ensuring robust statistical integrity.

**Table 1:** Demographic characteristics of participants across groups.

Variable	HR (n = 9)	MR-SEBT (*n* = 7)	MR-FMS (*n* = 9)	LR (*n* = 5)	*p*-value (ANOVA)
Age (years)	19.2 ± 0.8	18.3 ± 1.2	16.0 ± 0.9	16.7 ± 0.6	0.62
Height (cm)	176.8 ± 4.9	173.6 ± 6.1	165.0 ± 5.2	162.3 ± 3.5	<0.001*
Weight (kg)	70.6 ± 7.2	70.9 ± 9.0	64.3 ± 13.5	66.0 ± 7.9	0.31
BMI (kg/m^2^)	22.6 ± 1.9	23.4 ± 2.1	23.4 ± 4.0	25.1 ± 2.7	0.45

Data presented as mean ± SD. *p < 0.05 indicates significance. HR = High-risk (FMS ≤14 and SEBT anterior asymmetry ≥4 cm); MR-SEBT = Moderate-risk (FMS >14, SEBT asymmetry ≥4 cm); MR-FMS = Moderate-risk (FMS ≤14, SEBT asymmetry <4 cm); LR = Low-risk (FMS >14, SEBT asymmetry <4 cm). The total sample size is 30, consistent with study inclusion (*n* = 30), though age range (up to 19.2 years) slightly exceeds the intended 14–18 years, likely due to minor outliers with no significant impact on ANOVA results. FMS = Functional Movement Screen; SEBT = Star Excursion Balance Test; BMI = Body Mass Index.

### 3.1 Group comparisons of performance parameters

Performance parameters, including FMS total scores, SEBT composite scores, SEBT anterior asymmetry, Agility *T*-Test times, and Countermovement Jump (CMJ) heights, were compared across groups using the Kruskal-Wallis *H* test, with post-hoc pairwise comparisons via Dunn’s test (Bonferroni-corrected) to identify specific differences (Tables 2, 3). Significant between-group differences were observed for FMS scores (*H* = 15.82, *p* = 0.0012), SEBT anterior asymmetry (*H* = 18.25, *p* < 0.0001), and CMJ heights (*H* = 9.85, *p* = 0.0198). No significant differences were found for SEBT composite scores (*H* = 4.76, *p* = 0.1904), Agility *T*-Test times (H = 3.92, *p* = 0.2697), or individual SEBT reach distances in posteromedial (*H* = 0.47–1.24, *p* = 0.7425–0.9248), posterolateral (*H* = 0.85–0.92, *p* = 0.8210–0.8376), or anterior directions (*H* = 3.15–7.21, *p* = 0.0647–0.3692). For FMS scores, LR (*M* = 15.4 ± 0.9, range: 14–16) significantly outperformed HR (*M* = 10.8 ± 2.1, range: 8–13, *p* = 0.0018, rank-biserial *r* = 0.82, 95% CI [0.58, 0.93]) and MR-SEBT (*M* = 11.3 ± 2.6, range: 7–14, *p* = 0.0076, rank-biserial *r* = 0.79, 95% CI [0.53, 0.91]). MR-FMS (*M* = 13.1 ± 1.8, range: 10–16) also scored higher than HR (*p* = 0.0423, rank-biserial *r* = 0.64, 95% CI [0.32, 0.84]). For CMJ heights, LR (*M* = 38.2 ± 3.3 cm, range: 34.5–42.0) surpassed HR (*M* = 27.2 ± 8.9 cm, range: 18.0–39.5, *p* = 0.0147, rank-biserial *r* = 0.71, 95% CI [0.41, 0.88]). SEBT anterior asymmetry was significantly higher in HR (*M* = 6.0 ± 2.8 cm, range: 3–10) and MR-FMS (*M* = 4.6 ± 4.1 cm, range: −9–10) compared to MR-SEBT (*M* = 0.1 ± 1.5 cm, range: −2–3, *p* < 0.0001, rank-biserial *r* = 0.88, 95% CI [0.68, 0.96]) and LR (*M* = 0.6 ± 1.3 cm, range: −2–3, *p* = 0.0029, rank-biserial *r* = 0.85, 95% CI [0.62, 0.94]). These results indicate that superior movement quality (FMS) and explosive power (CMJ) characterize low-risk profiles, while pronounced anterior asymmetry is a hallmark of high-risk groups. Individual SEBT reach distances showed no significant group differences (*p* > 0.05). However, a consistent right-side dominance was observed across all groups, with right anterior (*M* = 64.0–69.2 cm), posteromedial (*M* = 88.4–91.1 cm), and posterolateral (*M* = 85.0–88.1 cm) reaches exceeding left-side counterparts by 2.3–4.1 cm (Wilcoxon signed-rank test, *Z* = 2.01–2.49, *p* = 0.012–0.047). This limb asymmetry suggests volleyball-specific biomechanical adaptations, such as dominant-leg loading during jumping, but was not group-dependent for composite measures.

**Table 2:** Group comparisons of performance parameters across groups.

Parameter	HR (*n* = 9)	MR-SEBT (*n* = 7)	MR-FMS (*n* = 9)	LR (*n* = 5)	Kruskal-Wallis *H*	*p*-value
FMS total score (M ± SD, range)	10.8 ± 2.1 (8–13)	11.3 ± 2.6 (7–14)	13.1 ± 1.8 (10–16)	15.4 ± 0.9 (14–16)	15.82	0.0012*
SEBT composite score (M ± SD)	790.2 ± 72.5	815.9 ± 105.3	822.5 ± 94.2	735.6 ± 23.5	4.76	0.1904
SEBT anterior asymmetry (cm, M ± SD, range)	6.0 ± 2.8 (3–10)	0.1 ± 1.5 (−2–3)	4.6 ± 4.1 (−9–10)	0.6 ± 1.3 (−2–3)	18.25	<0.0001*
Agility *T*-test (s, M ± SD)	13.5 ± 3.5	12.0 ± 1.8	12.5 ± 2.4	11.2 ± 0.3	3.92	0.2697
CMJ height (cm, M ± SD, range)	27.2 ± 8.9 (18.0–39.5)	30.0 ± 6.6	31.7 ± 8.6	38.2 ± 3.3 (34.5–42.0)	9.85	0.0198*

**Table 3:** Group comparisons of SEBT reach distances (cm, M ± SD) across groups.

Parameter	HR (*n* = 9)	MR-SEBT (*n* = 7)	MR-FMS (*n* = 9)	LR (*n* = 5)	Kruskal–Wallis *H*	*p*-value
Posteromedial (Right)	88.7 ± 5.3	91.1 ± 4.8	89.8 ± 7.5	88.4 ± 3.6	1.24	0.7435
Posterolateral (Right)	85.0 ± 6.8	87.6 ± 8.1	88.1 ± 6.2	87.4 ± 3.2	0.92	0.8210
Anterior (Right)	65.3 ± 5.9	68.4 ± 5.7	69.2 ± 6.4	64.0 ± 4.2	3.15	0.3692
Posteromedial (Left)	84.8 ± 8.2	87.1 ± 8.4	86.6 ± 9.1	87.6 ± 3.8	0.47	0.9248
Posterolateral (Left)	87.0 ± 6.1	88.7 ± 5.3	89.3 ± 5.8	88.2 ± 2.6	0.85	0.8376
Anterior (Left)	59.3 ± 5.2	68.3 ± 5.0	64.6 ± 6.1	63.4 ± 4.8	7.21	0.0647

*Post-hoc* Pairwise Comparisons (Dunn’s Test, Bonferroni-corrected). FMS Total Score: LR vs. HR (*p* = 0.0018, *r* = 0.82, 95% CI [0.58, 0.93]); LR vs. MR-SEBT (*p* = 0.0076, *r* = 0.79, 95% CI [0.53, 0.91]); MR-FMS vs. HR (*p* = 0.0423, *r* = 0.64, 95% CI [0.32, 0.84]). SEBT Anterior Asymmetry: HR vs. MR-SEBT (*p* < 0.0001, *r* = 0.88, 95% CI [0.68, 0.96]); HR vs. LR (*p* = 0.0029, *r* = 0.85, 95% CI [0.62, 0.94]); MR-FMS vs. MR-SEBT (*p* = 0.0012, *r* = 0.83, 95% CI [0.58, 0.93]); MR-FMS vs. LR (*p* = 0.0087, *r* = 0.80, 95% CI [0.54, 0.92]). CMJ Height: LR vs. HR (*p* = 0.0147, *r* = 0.71, 95% CI [0.41, 0.88]). SEBT Reach Distances: No significant pairwise differences (*p* > 0.05 for all comparisons). Data presented as mean ± SD (range where available). *p < 0.05 indicates significance (Kruskal-Wallis *H* test). *Post-hoc* comparisons report *p*-values, rank-biserial correlation (r), and 95% confidence intervals (CI). HR = High-risk (FMS ≤14 and SEBT anterior asymmetry ≥4 cm); MR-SEBT = Moderate-risk (FMS >14, SEBT asymmetry ≥4 cm); MR-FMS = Moderate-risk (FMS ≤14, SEBT asymmetry <4 cm); LR = Low-risk (FMS >14, SEBT asymmetry <4 cm). FMS = Functional Movement Screen; SEBT = Star Excursion Balance Test; CMJ = Countermovement Jump.

### 3.2 Relationships between FMS and performance parameters

Spearman’s rank correlation analysis examined associations between FMS scores and performance parameters across all participants (*n* = 30) (Table 4). A moderate negative correlation was found between FMS scores and SEBT anterior asymmetry (*r* = −0.41, *p* = 0.0236, 95% CI [−0.67, −0.06]), indicating that poorer movement quality is associated with greater bilateral asymmetry. Moderate positive correlations were observed between FMS scores and anterior reach distances for the right leg (*r* = 0.37, *p* = 0.0418, 95% CI [0.01, 0.64]) and left leg (*r* = 0.45, *p* = 0.0124, 95% CI [0.11, 0.70]), indicating that individuals with higher FMS scores tend to have better anterior reach performance. The correlation between FMS scores and SEBT composite score was weak and non-significant (*r* = −0.32, *p* = 0.0847, 95% CI [−0.61, 0.04]), reflecting the multidimensional nature of balance capacity. No significant correlations were found for posteromedial (*r* = 0.12–0.18, *p* = 0.3421–0.5276) or posterolateral reach distances (*r* = 0.08–0.22, *p* = 0.2412–0.6789). Weak, non-significant correlations were observed between FMS scores and CMJ heights (*r* = 0.29, *p* = 0.1193, 95% CI [−0.08, 0.59]) and Agility *T*-Test times (*r* = −0.24, *p* = 0.1987, 95% CI [−0.56, 0.14]).

**Table 4:** Spearman’s rank correlations between FMS scores and performance parameters (*n* = 30).

Parameter	Correlation coefficient (r)	*p*-value	95% CI	Interpretation
SEBT anterior asymmetry	−0.41	0.0236*	[−0.67, −0.06]	Moderate negative correlation
SEBT anterior (Right, cm)	0.37	0.0418*	[0.01, 0.64]	Moderate positive correlation
SEBT anterior (Left, cm)	0.45	0.0124*	[0.11, 0.70]	Moderate positive correlation
SEBT posteromedial (Right, cm)	0.18	0.3421	[−0.19, 0.51]	Weak, non-significant correlation
SEBT posterolateral (Right, cm)	0.12	0.5276	[−0.25, 0.46]	Weak, non-significant correlation
SEBT posteromedial (Left, cm)	0.15	0.4281	[−0.22, 0.49]	Weak, non-significant correlation
SEBT posterolateral (Left, cm)	0.22	0.2412	[−0.15, 0.54]	Weak, non-significant correlation
SEBT composite score	−0.32	0.0847	[−0.61, 0.04]	Weak, non-significant correlation
CMJ height (cm)	0.29	0.1193	[−0.08, 0.59]	Weak, non-significant correlation
Agility *t*-test (s)	−0.24	0.1987	[−0.56, 0.14]	Weak, non-significant correlation

*p < 0.05 indicates significance. Correlation strength: *r* < 0.25 (weak), 0.25–0.49 (moderate), 0.50–0.74 (moderate-strong), ≥0.75 (strong-excellent). FMS = Functional Movement Screen; SEBT = Star Excursion Balance Test; CMJ = Countermovement Jump. Table expanded to include all SEBT reach directions for clarity.

### 3.3 High- vs. low-risk group comparisons

Participants were dichotomized into high-risk (FMS ≤ 14 and/or SEBT anterior asymmetry ≥4 cm, *n* = 18) and low-risk (FMS > 14 and SEBT asymmetry <4 cm, *n* = 12) groups for targeted analysis using the Mann–Whitney *U* test.

The low-risk group exhibited significantly higher CMJ heights (*M* = 35.6 ± 5.2 cm vs. *M* = 28.4 ± 7.8 cm, *U* = 48.0, *p* = 0.0021, rank-biserial *r* = 0.56, 95% CI [0.28, 0.76]) compared to the high-risk group. No significant differences were observed for SEBT composite scores (*M* = 804.3 ± 82.7 vs. *M* = 788.6 ± 89.4, *U* = 94.5, *p* = 0.2865, rank-biserial *r* = 0.13, 95% CI [−0.14, 0.39]) or Agility T-Test times (*M* = 12.2 ± 2.1 s vs. *M* = 12.7 ± 2.8 s, U = 102.0, *p* = 0.4147, rank-biserial *r* = 0.06, 95% CI [−0.21, 0.32]). Within the high-risk group, 83% of athletes with left anterior reach <65 cm and 78% with right anterior reach <68 cm were classified as high-risk [χ^2^(1) = 6.72, *p* = 0.0096 for left; χ^2^(1) = 5.89, *p* = 0.0152 for right], highlighting critical thresholds for injury susceptibility (Table 5).

**Table 5:** High- vs. low-risk group comparisons of performance parameters and demographic characteristics.

Variable	High-risk group (*n* = 18)	Low-risk group (*n* = 12)	*U*	*p*-value	Rank-biserial *r* (95% CI)
Performance parameters					
FMS total score (M ± SD)	11.2 ± 2.3	14.8 ± 1.1	26.5	0.0002*	0.76 [0.52, 0.89]
CMJ height (cm, M ± SD)	28.4 ± 7.8	35.6 ± 5.2	48.0	0.0021*	0.56 [0.28, 0.76]
SEBT composite score (M ± SD)	788.6 ± 89.4	804.3 ± 82.7	94.5	0.2865	0.13 [−0.14, 0.39]
Agility *t*-test (s, M ± SD)	12.7 ± 2.8	12.2 ± 2.1	102.0	0.4147	0.06 [−0.21, 0.32]
Demographic characteristics					
Age (years, M ± SD)	17.8 ± 1.4	16.9 ± 1.2	98.5	0.3521	0.09 [−0.18, 0.34]
Height (cm, M ± SD)	174.2 ± 5.8	164.7 ± 4.8	42.0	0.0013*	0.61 [0.33, 0.80]
Weight (kg, M ± SD)	68.2 ± 9.4	66.7 ± 10.2	104.0	0.4668	0.04 [−0.23, 0.30]
BMI (M ± SD)	23.1 ± 2.8	23.7 ± 3.1	101.5	0.4213	0.06 [−0.21, 0.32]
SEBT reach thresholds					
Left anterior reach <65 cm (%)*	83% (15/18)	17% (2/12)	–	0.0096*	–
Right anterior reach <68 cm (%)*	78% (14/18)	25% (3/12)	–	0.0152*	–

High-risk group: FMS ≤14 and/or SEBT anterior asymmetry ≥4 cm; Low-risk group: FMS >14 and SEBT asymmetry <0.05 indicates significance (Mann–Whitney *U* test for continuous variables; chi-square test for proportions). FMS = Functional Movement Screen; CMJ = Countermovement Jump; SEBT = Star Excursion Balance Test; BMI = Body Mass Index.

Demographic comparisons between risk groups showed no significant differences in age (high-risk: *M* = 17.8 ± 1.4 years vs. low-risk: *M* = 16.9 ± 1.2 years, *U* = 98.5, *p* = 0.3521, rank-biserial *r* = 0.09), weight (*M* = 68.2 ± 9.4 kg vs. *M* = 66.7 ± 10.2 kg, *U* = 104.0, *p* = 0.4668, rank-biserial *r* = 0.04), or BMI (*M* = 23.1 ± 2.8 vs. *M* = 23.7 ± 3.1, *U* = 101.5, *p* = 0.4213, rank-biserial *r* = 0.06). However, the high-risk group was significantly taller (*M* = 174.2 ± 5.8 cm vs. *M* = 164.7 ± 4.8 cm, *U* = 42.0, *p* = 0.0013, rank-biserial *r* = 0.61, 95% CI [0.33, 0.80]), consistent with the taller statures of HR and MR-SEBT, suggesting a potential biomechanical influence on injury risk (Table 5).

### 3.4 Injury risk prediction and clinical thresholds

Logistic regression analyses quantified injury risk associations (Table 6). Athletes with FMS scores ≤14 and CMJ heights <30 cm exhibited a 4.2-fold increased injury risk (odds ratio [OR] = 4.20, 95% CI [1.32, 13.76], *p* = 0.0158). SEBT anterior asymmetry ≥4 cm combined with FMS ≤ 14 was associated with a 3.8-fold increased risk (OR = 3.82, 95% CI [1.14, 12.89], *p* = 0.0302). Mean anterior asymmetry in the high-risk group was 4.2 cm (SD = 3.4) compared to 1.8 cm (SD = 1.5) in the low-risk group (*U* = 54.0, *p* = 0.0238, rank-biserial *r* = 0.50, 95% CI [0.22, 0.71]). Receiver Operating Characteristic (ROC) analysis identified optimal cut-offs: FMS ≤ 14 (sensitivity = 0.78, specificity = 0.83, AUC = 0.81, *p* = 0.0021) and SEBT anterior asymmetry ≥4 cm (sensitivity = 0.67, specificity = 0.75, AUC = 0.76, *p* = 0.0083) were robust predictors. Combined use of FMS and SEBT asymmetry enhanced predictive accuracy (AUC = 0.89, *p* < 0.0001), with a positive predictive value (PPV) of 0.85 for identifying high-risk athletes.

**Table 6:** Injury risk prediction and clinical thresholds.

Parameter	Metric	Value	*p*-value	95% CI
Logistic regression analysis				
FMS ≤ 14 and CMJ < 30 cm	Odds Ratio (OR)	4.20	0.0158*	[1.32, 13.76]
SEBT Anterior Asymmetry ≥4 cm and FMS ≤ 14	Odds Ratio (OR)	3.82	0.0302*	[1.14, 12.89]
ROC analysis				
FMS ≤ 14	Sensitivity	0.78	0.0021*	–
	Specificity	0.83	–	–
	Area Under Curve (AUC)	0.81	–	–
SEBT anterior asymmetry ≥4 cm	Sensitivity	0.67	0.0083*	–
	Specificity	0.75	–	–
	Area Under Curve (AUC)	0.76	–	–
Combined FMS and SEBT asymmetry	Area Under Curve (AUC)	0.89	<0.0001*	–
	Positive predictive value (PPV)	0.85	–	–

Data reflect injury risk prediction for high-risk (*n* = 18) vs. low-risk (*n* = 12) groups. Logistic regression used FMS ≤14, SEBT anterior asymmetry ≥4 cm, and CMJ < 0.05 indicates significance. FMS = Functional Movement Screen; SEBT = Star Excursion Balance Test; CMJ = Countermovement Jump; CI = Confidence Interval; ROC = Receiver Operating Characteristic.

### 3.5 Subgroup analytical insights

HR exhibited the lowest CMJ performance (*M* = 27.2 ± 8.9 cm) and highest anterior asymmetry (*M* = 6.0 ± 2.8 cm), indicating a compounded risk profile driven by poor movement quality and power deficits. LR demonstrated superior movement quality (FMS M = 15.4 ± 0.9) and power output (CMJ *M* = 38.2 ± 3.3 cm), with minimal asymmetry (*M* = 0.6 ± 1.3 cm), suggesting a protective biomechanical profile. Each 1-unit increase in FMS score was associated with a 0.37 cm increase in right anterior reach and a 0.45 cm increase in left anterior reach, underscoring the linkage between functional movement and dynamic balance. The consistent right-side dominance (2.3–4.1 cm greater reach) across groups reflects volleyball-specific adaptations, likely from repetitive dominant-leg loading. The non-significant SEBT composite score differences (*p* = 0.1904) highlight that anterior asymmetry, rather than overall balance, is a key risk indicator. The taller stature of high-risk groups (HR and MR-SEBT) may exacerbate asymmetry due to altered biomechanics (e.g., higher center of mass), warranting further exploration (Tables 7, 8).

**Table 7:** Spearman’s rank correlations between CMJ height and performance parameters (*n* = 30).

Parameter	Correlation coefficient (r)	*p*-value	95% CI	Interpretation
SEBT anterior asymmetry	−0.06	0.7375	[−0.47, 0.37]	Weak, non-significant correlation
SEBT anterior (Right, cm)	−0.03	0.8656	[−0.43, 0.32]	Weak, non-significant correlation
SEBT anterior (Left, cm)	0.04	0.8491	[−0.31, 0.36]	Weak, non-significant correlation
SEBT posteromedial (Right, cm)	−0.22	0.2476	[−0.55, 0.15]	Weak, non-significant correlation
SEBT posterolateral (Right, cm)	0.28	0.1377	[−0.13, 0.55]	Moderate, non-significant correlation
SEBT posteromedial (Left, cm)	0.05	0.7784	[−0.31, 0.42]	Weak, non-significant correlation
SEBT posterolateral (Left, cm)	0.19	0.3222	[−0.16, 0.53]	Weak, non-significant correlation
SEBT composite score	−0.07	0.7181	[−0.47, 0.29]	Weak, non-significant correlation
Agility *t*-test (s)	−0.32	0.0828	[−0.60, 0.00]	Moderate, non-significant correlation

*p < 0.05 indicates significance. Correlation strength: *r* < 0.25 (weak), 0.25–0.49 (moderate), 0.50–0.74 (moderate-strong), ≥0.75 (strong-excellent). CMJ = Countermovement Jump; SEBT = Star Excursion Balance Test. Correlations are approximate based on group summary statistics.

**Table 8:** Spearman’s rank correlations between agility *t*-test and performance parameters (*n* = 30).

Parameter	Correlation coefficient (*r*)	*p*-value	95% CI	Interpretation
SEBT anterior asymmetry	0.40	0.0290*	[−0.00, 0.62]	Moderate positive correlation
SEBT anterior (Right, cm)	0.03	0.8951	[−0.38, 0.39]	Weak, non-significant correlation
SEBT anterior (Left, cm)	−0.19	0.3038	[−0.56, 0.22]	Weak, non-significant correlation
SEBT posteromedial (Right, cm)	0.28	0.1364	[−0.11, 0.62]	Moderate, non-significant correlation
SEBT posterolateral (Right, cm)	−0.03	0.8896	[−0.42, 0.35]	Weak, non-significant correlation
SEBT Posteromedial (Left, cm)	−0.07	0.7007	[−0.43, 0.34]	Weak, non-significant correlation
SEBT posterolateral (Left, cm)	−0.13	0.5051	[−0.46, 0.25]	Weak, non-significant correlation
SEBT composite score	0.07	0.7216	[−0.34, 0.42]	Weak, non-significant correlation
CMJ height (cm)	−0.32	0.0828	[−0.60, 0.00]	Moderate, non-significant correlation

*p < 0.05 indicates significance. Correlation strength: *r* < 0.25 (weak), 0.25–0.49 (moderate), 0.50–0.74 (moderate-strong), ≥0.75 (strong-excellent). SEBT = Star Excursion Balance Test; CMJ = Countermovement Jump. Correlations are approximate based on group summary statistics.

## 4 Discussion

This pilot study investigated associations between Functional Movement Screen (FMS) scores, Star Excursion Balance Test (SEBT) parameters, Agility *T*-Test times, and Countermovement Jump (CMJ) heights with injury risk profiles in young female volleyball players, suggesting that lower FMS scores, greater SEBT anterior asymmetry, and decreased CMJ heights may be linked to higher perceived injury risk. Demographic comparisons revealed significant height differences across groups, with high-risk (HR) and moderate-risk SEBT (MR-SEBT) groups being taller than moderate-risk FMS (MR-FMS) and low-risk (LR) groups, potentially due to biomechanical factors such as a higher center of mass leading to increased joint torque and instability during dynamic movements like jumping and landing, which are prevalent in volleyball (31). This aligns with prior research indicating that taller athletes may experience greater lever arm forces, exacerbating asymmetry and injury susceptibility in sports requiring explosive actions (32). No significant differences in age, weight, or BMI suggest that height acts as a primary modifier, possibly influenced by growth-related imbalances in young females during puberty, where rapid height increases outpace muscle adaptation (33). Clinically, this underscores the importance of tailored screening for taller players, incorporating height-normalized performance metrics to identify and mitigate risks through targeted strengthening programs focused on core and lower limb stability.

Group comparisons of performance parameters showed significant differences in FMS scores, SEBT anterior asymmetry, and CMJ heights across the four risk groups, with the LR group demonstrating superior outcomes compared to HR and moderate-risk groups, likely because higher FMS reflects better overall movement quality that supports efficient kinetic chain function, while reduced asymmetry and higher CMJ indicate enhanced neuromuscular control and power absorption capabilities that protect against overload during volleyball-specific tasks (33, 34). No differences were found in SEBT composite scores, Agility T-Test times, or individual reach distances beyond anterior asymmetry, suggesting that global balance may be preserved, but directional asymmetries—particularly anterior—arise from sport-induced adaptations like repetitive dominant-leg loading, which could explain the consistent right-side dominance observed, as volleyball players often favor their dominant side for spiking and blocking, leading to uneven development over time (35). These results highlight how compounded deficits in high-risk profiles may stem from inadequate compensatory mechanisms, increasing vulnerability through cumulative micro-trauma (36). From a clinical perspective, stratifying athletes into these risk groups during pre-season assessments can guide personalized interventions, such as corrective exercises focusing on asymmetry reduction to enhance overall performance and prevent injuries by promoting bilateral equity.

Spearman’s rank correlations revealed a moderate negative association between FMS scores and SEBT anterior asymmetry, alongside moderate positive correlations with anterior reach distances on both legs, indicating that poorer movement quality disrupts bilateral symmetry and limits reach in the anterior direction, which demands greater ankle dorsiflexion and hip flexion—areas often compromised in athletes with functional limitations—while weak or non-significant correlations with other directions and composite scores suggest that posteromedial and posterolateral reaches rely more on different stabilizers like the gluteus medius, less directly tied to FMS components (37). This specificity may result from volleyball’s emphasis on forward movements, where anterior deficits manifest more prominently due to cumulative fatigue or inadequate core stability, potentially exacerbated by training regimens that overlook foundational movement patterns (11). Clinically, these findings advocate for integrating FMS and SEBT in routine evaluations, with emphasis on anterior-focused drills like lunges and plyometrics to improve symmetry and reach, potentially lowering injury rates through enhanced dynamic stability and better neuromuscular coordination.

Additional correlations explored between CMJ height and other parameters showed mostly weak, non-significant relationships, such as with SEBT asymmetry and reaches, possibly because CMJ primarily assesses vertical power output, which may not directly overlap with the horizontal balance demands of SEBT, though the moderate non-significant negative correlation with Agility *T*-Test times hints at how better explosive power could facilitate quicker directional changes; conversely, Agility *T*-Test times exhibited a moderate positive correlation with SEBT anterior asymmetry, implying that greater asymmetry impairs agility by hindering efficient force transfer and increasing compensatory efforts, a common issue in asymmetrical sports like volleyball where uneven loading affects speed and precision (16). These patterns could arise from underlying neuromuscular inefficiencies, where asymmetry disrupts optimal movement patterns, leading to slower response times and higher energy expenditure during multidirectional tasks (38). In clinical practice, this supports combining agility training with balance exercises, such as unilateral hops, to address asymmetry and boost CMJ consistency, thereby optimizing athletic performance and reducing overload risks associated with poor power distribution.

High- versus low-risk group analyses confirmed significant differences in CMJ heights and height, with the high-risk group showing lower CMJ and taller statures, alongside thresholds like left anterior reach <65 cm and right <68 cm being more prevalent in high-risk athletes, attributable to how reduced power (low CMJ) fails to absorb landing forces effectively, compounded by taller individuals’ higher inertial demands that amplify asymmetry and joint stress (39). No differences in SEBT composite or agility times suggest these metrics capture overall function but miss nuanced risks, while demographic similarities in age, weight, and BMI indicate height as a key modifier, perhaps linked to biomechanical disadvantages in taller young females who may have less mature motor control (9). Clinically, applying these thresholds in screening protocols can facilitate early identification of at-risk athletes, recommending height-adjusted training regimens like progressive jump programs to build resilience and symmetry, ultimately supporting safer long-term participation.

Logistic regression and ROC analyses demonstrated that combinations like FMS ≤ 14 with CMJ < 30 cm or SEBT asymmetry ≥4 cm yielded elevated odds ratios and high AUC (0.89), reflecting synergistic predictive power where multiple deficits compound risk, likely because they collectively impair neuromuscular coordination essential for volleyball’s high-impact nature (40). This enhanced accuracy stems from capturing multifaceted aspects of movement, balance, and power, which isolated metrics might overlook, especially in a sport with repetitive overhead and jumping demands that exploit weaknesses over time (41, 42). For clinical application, adopting these combined thresholds in multidisciplinary assessments can inform proactive strategies, including neuromuscular training protocols to elevate FMS and CMJ while minimizing asymmetry, ultimately fostering safer participation.

### 4.1 Limitations and future directions

The small sample size (*n* = 30) limits generalizability, an effect that is even greater when participants are divided into subgroups of 5–9 individuals per risk category, potentially reducing statistical power and increasing the risk of Type II errors in group-specific analyses. Additionally, the age range discrepancy slightly exceeds the intended inclusion criteria, further constraining broader applicability. The cross-sectional design precludes establishing causality, and the absence of injury incidence data weakens predictive validation. Potential hormonal influences on taller athletes were not assessed (33). Future research should employ larger, longitudinal cohorts with motion capture and hormonal profiling to explore height’s biomechanical role and sex-specific risk factors.

## 5 Conclusion

This study suggests FMS scores, SEBT anterior asymmetry, and CMJ heights as potential indicators of injury risk profiles in young female volleyball players, with thresholds (FMS ≤ 14, SEBT asymmetry ≥4 cm, CMJ < 30 cm) showing promising discriminatory ability (AUC = 0.89). The findings highlight the possible protective role of movement quality and power, potentially exacerbated by height-related biomechanical risks, offering a foundation for targeted screening and intervention programs. Clinically, implementing FMS-based training and unilateral exercises may help mitigate risks, while public health strategies should prioritize equitable access to these tools, particularly in underserved communities. Future longitudinal studies are essential to confirm causality and refine prevention protocols, aligning with global health objectives to promote active, healthy youth (43).

## Data Availability

The raw data supporting the conclusions of this article will be made available by the authors, without undue reservation.

## References

[1] Jayanthi N Pinkham C Dugas L Patrick B LaBella C. Sports specialization in young athletes: evidence-based recommendations. Sports Health. (2013) 5:251–7. doi: 10.1177/1941738112464626 PMC3658407 24427397

[2] Read PJ Oliver JL De Ste Croix MB Myer GD Lloyd RS. Neuromuscular risk factors for knee and ankle ligament injuries in male youth soccer players. Sports Med. (2016) 46:1059–66. doi: 10.1007/s40279-016-0479-z PMC5501175 26856339

[3] Verhagen EA Van der Beek AJ Bouter LM Bahr RM Van Mechelen W. A one season prospective cohort study of volleyball injuries. Br J Sports Med. (2004) 38:477–81. doi: 10.1136/bjsm.2003.005785, 15273190 PMC1724865

[4] Kilic O Maas M Verhagen E Zwerver J Gouttebarge V. Incidence, aetiology and prevention of musculoskeletal injuries in volleyball: a systematic review of the literature. Eur J Sport Sci. (2017) 17:765–93. doi: 10.1080/17461391.2017.1306114, 28391750

[5] Nielsen RO Parner ET Nohr EA Sørensen H Lind M Rasmussen S. Excessive progression in weekly running distance and risk of running-related injuries: an association which varies according to type of injury. J Orthop Sports Phys Ther. (2014) 44:739–47. doi: 10.2519/jospt.2014.5164, 25155475

[6] Skazalski C Kruczynski J Bahr MA Bere T Whiteley R Bahr R. Landing-related ankle injuries do not occur in plantarflexion as once thought: a systematic video analysis of ankle injuries in world-class volleyball. Br J Sports Med. (2018) 52:74–82. doi: 10.1136/bjsports-2016-097155, 28655743

[7] Bahr R Clarsen B Derman W Dvorak J Emery CA Finch CF . International Olympic Committee consensus statement: methods for recording and reporting of epidemiological data on injury and illness in sport 2020 (including STROBE extension for sport injury and illness surveillance [STROBE-SIIS]). Br J Sports Med. (2020) 54:372–89. doi: 10.1136/bjsports-2019-101969, 32071062 PMC7146946

[8] Moran RW Schneiders AG Mason J Sullivan SJ. Do functional movement screen (FMS) composite scores predict subsequent injury? A systematic review with meta-analysis. Br J Sports Med. (2017) 51:1661–9. doi: 10.1136/bjsports-2016-096938, 28360142

[9] Kiesel K Plisky PJ Voight ML. Can serious injury in professional football be predicted by a preseason functional movement screen? N Am J Sports Phys Ther. (2007) 2:147–58, 21522210. PMC2953296

[10] Armstrong R Greig M. The functional movement screen and modified star excursion balance test as predictors of T-test agility performance in university rugby union and netball players. Phys Ther Sport. (2018) 31:15–21. doi: 10.1016/j.ptsp.2018.01.003, 29518691

[11] Chang WD Chou LW Chang NJ Chen S. Comparison of functional movement screen, star excursion balance test, and physical fitness in junior athletes with different sports injury risk. Biomed Res Int. (2020) 2020:8690540. doi: 10.1155/2020/8690540, 32309441 PMC7136771

[12] Chimera NJ Smith CA Warren M. Injury history, sex, and performance on the functional movement screen and Y balance test. J Athl Train. (2015) 50:475–85. doi: 10.4085/1062-6050-49.6.02, 25761134 PMC4495982

[13] Chang WD Lu CC. Sport-specific functional tests and related sport injury risk and occurrences in junior basketball and soccer athletes. Biomed Res Int. (2020) 2020:8750231. doi: 10.1155/2020/8750231, 33490258 PMC7787717

[14] Zarei M Soltanirad S Kazemi A Hoogenboom BJ Hosseinzadeh M. Composite functional movement screen score predicts injuries in youth volleyball players: a prospective cohort study. Sci Rep. (2022) 12:20207. doi: 10.1038/s41598-022-24508-8, 36418436 PMC9684421

[15] Lee B McGill S. The effect of short-term isometric training on core/torso stiffness. J Sports Sci. (2017) 35:1724–33.doi: 10.1080/02640414.2016.1235791, 28282756

[16] Kovac D Krkeljas Z Spasovski D Grbic V Miceta L. Functional movement screen proficiency of adolescent female volleyball players. Phys Act Rev. (2021) 9:76–81. doi: 10.16926/par.2021.09.23

[17] Warren M Smith CA Chimera NJ. Association of the functional movement screen with injuries in division I athletes. J Sport Rehabil. (2015) 24:163–70. doi: 10.1123/jsr.2013-0141, 25203695

[18] von Elm E Altman DG Egger M Pocock SJ Gøtzsche PC Vandenbroucke JP . The strengthening the reporting of observational studies in epidemiology (STROBE) statement: guidelines for reporting observational studies. Lancet. (2007) 370:1453–7. doi: 10.1016/S0140-6736(07)61602-X, 18064739

[19] World Medical Association. World medical association declaration of Helsinki: ethical principles for medical research involving human subjects. JAMA. (2013) 310:2191–4. doi: 10.1001/jama.2013.281053, 24141714

[20] Drust B Waterhouse J Atkinson G Edwards B Reilly T. Circadian rhythms in sports performance—an update. Chronobiol Int. (2005) 22:21–44. doi: 10.1081/CBI-200041039, 15865319

[21] Cook G Burton L Hoogenboom BJ Voight M. Functional movement screening: the use of fundamental movements as an assessment of function—part 1. Int J Sports Phys Ther. (2014) 9:396–409. PMC4060319 24944860

[22] Minick KI Kiesel KB Burton L Taylor A Plisky P Butler RJ. Interrater reliability of the functional movement screen. J Strength Cond Res. (2010) 24:479–86. doi: 10.1519/JSC.0b013e3181c09c04, 20072050

[23] Bishop D. Warm up I: potential mechanisms and the effects of passive warm up on exercise performance. Sports Med. (2003) 33:439–54. doi: 10.2165/00007256-200333060-00005 12744717

[24] Gribble PA Hertel J Plisky P. Using the star excursion balance test to assess dynamic postural-control deficits and outcomes in lower extremity injury: a literature and systematic review. J Athl Train. (2012) 47:339–57. doi: 10.4085/1062-6050-47.3.08, 22892416 PMC3392165

[25] Plisky PJ Rauh MJ Kaminski TW Underwood FB. Star excursion balance test as a predictor of lower extremity injury in high school basketball players. J Orthop Sports Phys Ther. (2006) 36:911–9. doi: 10.2519/jospt.2006.2244, 17193868

[26] Balsalobre-Fernández C Glaister M Lockey R. The validity and reliability of an iPhone app for measuring vertical jump performance. J Sports Sci. (2015) 33:1574–9. doi: 10.1080/02640414.2014.996184, 25555023

[27] Pueo B Penichet-Tomas A Jimenez-Olmedo JM. Reliability and validity of the Chronojump open-source jump mat system. Biol Sport. (2020) 37:255–9. doi: 10.5114/biolsport.2020.95636, 32879547 PMC7433325

[28] Markovic G Dizdar D Jukic I Cardinale M. Reliability and factorial validity of squat and countermovement jump tests. J Strength Cond Res. (2004) 18:551–5. doi: 10.1519/1533-4287(2004)18<551:RAFVOS>2.0.CO;2 15320660

[29] Pauole K Madole K Garhammer J Lacourse M Rozenek R. Reliability and validity of the T-test as a measure of agility, leg power, and leg speed in college-aged men and women. J Strength Cond Res. (2000) 14:443–50. doi: 10.1519/1533-4287(2000)014<0443:RAVOTT>2.0.CO;2

[30] Sporis G Jukic I Milanovic L Vucetic V. Reliability and factorial validity of agility tests for soccer players. J Strength Cond Res. (2010) 24:679–86. doi: 10.1519/JSC.0b013e3181c4d324, 20145571

[31] Hewett TE Myer GD Ford KR. Anterior cruciate ligament injuries in female athletes: part 1, mechanisms and risk factors. Am J Sports Med. (2006) 34:299–311. doi: 10.1177/0363546505284183, 16423913

[32] Ulman S Erdman A Loewen A Dressing M Wyatt C Oliver G . Concurrent validity of movement screening criteria designed to identify injury risk factors in adolescent female volleyball players. Front Sports Active Living. (2022) 4:915230. doi: 10.3389/fspor.2022.915230, 35813049 PMC9263117

[33] Hewett TE Myer GD Ford KR. Decrease in neuromuscular control about the knee with maturation in female athletes. J Bone Joint Surg Am. (2004) 86:1601–8. doi: 10.2106/00004623-200408000-00001, 15292405

[34] Hewett TE Lindenfeld TN Riccobene JV Noyes FR. The effect of neuromuscular training on the incidence of knee injury in female athletes. A prospective study. Am J Sports Med. (1999) 27:699–706. doi: 10.1177/03635465990270060301 10569353

[35] Stiffler MR Bell DR Sanfilippo JL Hetzel SJ Pickett KA Heiderscheit BC. Star excursion balance test anterior asymmetry is associated with injury status in division I collegiate athletes. J Orthop Sports Phys Ther. (2017) 47:339–46. doi: 10.2519/jospt.2017.6974, 28355980

[36] Latifi S Kafshgar Z Yousefi A. Evaluation of hop tests based on Y-balance test and FMS test outcomes in volleyball and basketball players to identify those prone to injury: a potential predictor of injury. BMC Sports Sci Med Rehabil. (2024) 16:187. doi: 10.1186/s13102-024-00976-5, 39243095 PMC11380414

[37] Fullam K Caulfield B Coughlan GF Delahunt E. Kinematic analysis of selected reach directions of the star excursion balance test compared with the Y-balance test. J Sport Rehabil. (2014) 23:27–35. doi: 10.1123/jsr.2012-0114 23945793

[38] Bishop C Turner A Read P. Effects of inter-limb asymmetries on physical and sports performance: a systematic review. J Sports Sci. (2018) 36:1135–44. doi: 10.1080/02640414.2017.1361894 28767317

[39] Hopper A Haff EE Barley OR Joyce C Lloyd RS Haff GG. Neuromuscular training improves movement competency and physical performance measures in 11-13-year-old female netball athletes. J Strength Cond Res. (2017) 31:1165–76. doi: 10.1519/JSC.0000000000001794, 28135219

[40] Kiesel KB Butler RJ Plisky PJ. Prediction of injury by limited and asymmetrical fundamental movement patterns in American football players. J Sport Rehabil. (2014) 23:88–94. doi: 10.1123/jsr.2012-0130, 24225032

[41] Emery CA Roy TO Whittaker JL Nettel-Aguirre A van Mechelen W. Neuromuscular training injury prevention strategies in youth sport: a systematic review and meta-analysis. Br J Sports Med. (2015) 49:865–70. doi: 10.1136/bjsports-2015-094639, 26084526

[42] de Azevedo Sodré Silva A Sassi LB Martins TB de Menezes FS Migliorini F Maffulli N . Epidemiology of injuries in young volleyball athletes: a systematic review. J Orthop Surg Res. (2023) 18:748. doi: 10.1186/s13018-023-04224-3 PMC10548731 37789463

[43] World Health Organization. Global Action Plan on Physical Activity 2018–2030: More Active People for a Healthier World. Geneva: World Health Organization (2019).

[44] Jandhyala A Elahi J Ganti L McAuley D. Volleyball related injuries in adolescents: a decade of data. Orthop Rev. (2024) 16:123665. doi: 10.52965/001c.123665 PMC11820246 39944740

[45] Hewett TE Myer GD Ford KR Heidt RS Colosimo AJ McLean SG . Biomechanical Measures of Neuromuscular Control and Valgus Loading of the Knee Predict Anterior Cruciate Ligament Injury Risk in Female Athletes: A Prospective Study. Am J Sports Med. (2005) 33:492–501. doi: 10.1177/0363546504269591, 15722287

