# Correction: Predicting injury risk in young female volleyball players through movement and jump assessments

**DOI:** 10.3389/fpubh.2026.2007172

**Published:** 2026-10-07

**Authors:** Mustafa Erol, Fatma Gozlukaya Girginer, Sinan Seyhan, Gorkem Acar, Gunay Cerit, Meliha Uzun, Caglar Soylu

**Affiliations:** 1 Faculty of Sports Sciences, Department of Exercise and Sport Sciences, Istanbul Rumeli University, Istanbul, Türkiye; 2 Faculty of Sport Sciences, Pamukkale University, Denizli, Türkiye; 3 Faculty of Sport Sciences, Department of Coaching Education, Manisa Celal Bayar University, Manisa, Türkiye; 4 Department of Sport Science, Institute of Graduate Education, Manisa Celal Bayar University, Manisa, Türkiye; 5 Department of Physical Education and Sports, School of Physical Education and Sports, Sirnak University, Sirnak, Türkiye; 6 School of Physical Education and Sports, Department of Coaching Education, Sirnak University, Sirnak, Türkiye; 7 Gülhane Faculty of Physiotherapy and Rehabilitation, University of Health Sciences, Ankara, Türkiye

**Keywords:** injury prevention, volleyball, female athletes, functional movement screen, injury risk assessment

In the published article, there were errors in the reference list. The affected references and their corrections are listed below.

The reference for citation 1 was erroneously written as “Jayanthi N, Pinkham C, Dugas L, Patrick B, LaBella C. Sports specialization in young athletes: evidence-based recommendations. *Sports Health*. (2015) 5:251–7. doi: 10.1177/1941738112464626”. It should be “Jayanthi N, Pinkham C, Dugas L, Patrick B, LaBella C. Sports specialization in young athletes: evidence-based recommendations. *Sports Health*. (2013) 5:251–7. doi: 10.1177/1941738112464626”.

The reference for citation 2 was erroneously written as “Read PJ, Oliver JL, De Ste Croix MB, Myer GD, Lloyd RS. Neuromuscular risk factors for knee and ankle ligament injuries in male youth soccer players. *Sports Med*. (2019) 49:219–36. doi: 10.1007/s40279-018-1003-1”. It should be “Read PJ, Oliver JL, De Ste Croix MB, Myer GD, Lloyd RS. Neuromuscular risk factors for knee and ankle ligament injuries in male youth soccer players. *Sports Med*. (2016) 46:1059–66. doi: 10.1007/s40279-016-0479-z”.

The reference for citation 4 was erroneously written as “Zarei M, Norasteh AA, Asadi A. Prevalence and risk factors of musculoskeletal injuries among elite volleyball players. *Int J Sports Med*. (2020) 41:845–52. doi: 10.1055/a-1182-3456”. It should be “Kilic O, Maas M, Verhagen E, Zwerver J, Gouttebarge V. Incidence, aetiology and prevention of musculoskeletal injuries in volleyball: a systematic review of the literature. *Eur J Sport Sci*. (2017) 17:765–93. doi: 10.1080/17461391.2017.1306114”.

The reference for citation 5 was erroneously written as “Neilsen RO, Parner ET, Nohr EA, Sørensen H. Excessive progression in weekly running distance and risk of running-related injuries: an association which varies according to type of injury. *J Orthop Sports Phys Ther*. (2020) 50:563–71. doi: 10.2519/jospt.2020.9400”. It should be “Nielsen RO, Parner ET, Nohr EA, Sørensen H, Lind M, Rasmussen S. Excessive progression in weekly running distance and risk of running-related injuries: an association which varies according to type of injury. *J Orthop Sports Phys Ther*. (2014) 44:739–47. doi: 10.2519/jospt.2014.5164”.

The reference for citation 6 was erroneously written as “Bere T, Mok KM, Krosshaug T, Bahr R. A systematic video analysis of injuries in professional volleyball: mechanisms and prevention strategies. *Scand J Med Sci Sports*. (2023) 33:456–65. doi: 10.1111/sms.14289”. It should be “Skazalski C, Kruczynski J, Bahr MA, Bere T, Whiteley R, Bahr R. Landing-related ankle injuries do not occur in plantarflexion as once thought: a systematic video analysis of ankle injuries in world-class volleyball. *Br J Sports Med*. (2018) 52:74–82. doi: 10.1136/bjsports-2016-097155”.

The reference for citation 8 was erroneously written as “Linek P, Saulicz E, Kuszewski M, Wolny T. Predictive value of the functional movement screen for injury in athletes: a systematic review. J Strength Cond Res. (2016) 30:2330–7. doi: 10.1519/JSC.0000000000001318”. It should be “Moran RW, Schneiders AG, Mason J, Sullivan SJ. Do functional movement screen (FMS) composite scores predict subsequent injury? A systematic review with meta-analysis. *Br J Sports Med*. (2017) 51:1661–9. doi: 10.1136/bjsports-2016-096938”.

The reference for citation 9 was erroneously written as “Wu G, Liu Y, Zhang L. Limb dominance and injury risk in volleyball: a biomechanical analysis. *J Biomech*. (2023) 150:111498. doi: 10.1016/j.jbiomech.2023.111498”. It should be “Kiesel K, Plisky PJ, Voight ML. Can serious injury in professional football be predicted by a preseason functional movement screen? *N Am J Sports Phys Ther*. (2007) 2:147–58.”

The reference for citation 10 was erroneously written as “Armstrong R, Greig M. The functional movement screen and T-test: a battery for assessing movement and agility in team sports. *J Sports Sci*. (2018) 36:1699–705. doi: 10.1080/02640414.2017.1413643”. It should be “Armstrong R, Greig M. The functional movement screen and modified star excursion balance test as predictors of T-test agility performance in university rugby union and netball players. *Phys Ther Sport*. (2018) 31:15–21. doi: 10.1016/j.ptsp.2018.01.003”.

The reference for citation 11 was erroneously written as “Chang WD, Chou LW, Lu YC. Functional movement screen as a predictor of sports injury risk in adolescent athletes. *J Athl Train*. (2020) 55:245–51. doi: 10.4085/1062-6050-44-19”. It should be “Chang WD, Chou LW, Chang NJ, Chen S. Comparison of functional movement screen, star excursion balance test, and physical fitness in junior athletes with different sports injury risk. *Biomed Res Int*. (2020) 2020:8690540. doi: 10.1155/2020/8690540”.

The reference for citation 12 was erroneously written as “Chimera NJ, Warren M, Smith CA. The star excursion balance test as a measure of lower extremity performance and injury risk. *J Orthop Sports Phys Ther*. (2015) 45:428–35. doi: 10.2519/jospt.2015.5404”. It should be “Chimera NJ, Smith CA, Warren M. Injury history, sex, and performance on the functional movement screen and Y balance test. *J Athl Train*. (2015) 50:475–85. doi: 10.4085/1062-6050-49.6.02”.

The reference for citation 13 was erroneously written as “Caswell SV, Cortes N, Chinn L, Keene K. The relationship between agility and injury risk in adolescent American football players: a multivariate analysis. *J Athl Train*. (2016) 51:718–24. doi: 10.4085/1062-6050-51.9.04”. It should be “Chang WD, Lu CC. Sport-specific functional tests and related sport injury risk and occurrences in junior basketball and soccer athletes. *Biomed Res Int*. (2020) 2020:8750231. doi: 10.1155/2020/8750231”.

The reference for citation 14 was erroneously written as “Larruskain G, Lekue JA, Diaz N, Odriozola A, Gil SM. Functional movement screen (FMS) as a predictor of injuries in young volleyball players: a prospective cohort study. *Int J Sports Med*. (2022) 44:197–204. doi: 10.1055/a-1963-7426”. It should be “Zarei M, Soltanirad S, Kazemi A, Hoogenboom BJ, Hosseinzadeh M. Composite functional movement screen score predicts injuries in youth volleyball players: a prospective cohort study. *Sci Rep*. (2022) 12:20207. doi: 10.1038/s41598-022-24508-8”.

The reference for citation 15 was erroneously written as “Lee BC, McGill SM. The effect of short-term isometric training on core/torso stiffness. *J Strength Cond Res*. (2020) 34:1519–26. doi: 10.1519/JSC.0000000000003609”. It should be “Lee B, McGill S. The effect of short-term isometric training on core/torso stiffness. *J Sports Sci*. (2017) 35:1724–33. doi: 10.1080/02640414.2016.1235791”.

The reference for citation 16 was erroneously written as “Smith RL, McGuine TA, Brooks MA. Agility deficits as injury predictors in adolescent athletes. Clin J Sport Med. (2023) 33:134–40. doi: 10.1097/JSM.0000000000001089”. It should be “Kovac D, Krkeljas Z, Spasovski D, Grbic V, Miceta L. Functional movement screen proficiency of adolescent female volleyball players. *Phys Act Rev*. (2021) 9:76–81. doi: 10.16926/par.2021.09.23”.

The reference for citation 17 was erroneously written as “Warren M, Smith CA, Chimera NJ. Composite functional movement screen score predicts injuries in youth volleyball players: a prospective cohort study. *J Sport Rehabil*. (2015) 24:163–70. doi: 10.1123/jsr.2013-0141”. It should be “Warren M, Smith CA, Chimera NJ. Association of the functional movement screen with injuries in division I athletes. *J Sport Rehabil*. (2015) 24:163–70. doi: 10.1123/jsr.2013-0141”.

The reference for citation 23 was erroneously written as “Bishop D. Warm up I: potential mechanisms and the effects of passive warm up on exercise performance. *Sports Med*. (2003) 33:439–54. doi: 10.2165/00007256-200333070-00005”. It should be “Bishop D. Warm up I: potential mechanisms and the effects of passive warm up on exercise performance. *Sports Med*. (2003) 33:439–54. doi: 10.2165/00007256-200333060-00005”.

The reference for citation 28 was erroneously written as “Markovic G, Dizdar D, Jukic I, Cardinale M. Reliability and factorial validity of squat and countermovement jump tests. *J Strength Cond Res*. (2004) 18:551–5. doi: 10.1519/1533-4287(2004)18”. It should be “Markovic G, Dizdar D, Jukic I, Cardinale M. Reliability and factorial validity of squat and countermovement jump tests. *J Strength Cond Res*. (2004) 18:551–5. doi: 10.1519/1533-4287(2004)18 < 551:RAFVOS>2.0.CO;2”.

The reference for citation 29 was erroneously written as “Pauole K, Madole K, Garhammer J, Lacourse M, Rozenek R. Reliability and validity of the T-test as a measure of agility, leg power, and leg speed in college-aged men and women. *J Strength Cond Res*. (2000) 14:443–50. doi: 10.1519/1533-4287(2000)014 < >2.0.CO;2”. It should be “Pauole K, Madole K, Garhammer J, Lacourse M, Rozenek R. Reliability and validity of the T-test as a measure of agility, leg power, and leg speed in college-aged men and women. *J Strength Cond Res*. (2000) 14:443–50. doi: 10.1519/1533-4287(2000)014 < 0443:RAVOTT>2.0.CO;2”.

The reference for citation 31 was erroneously written as “Hewett TE, Ford KR, Myer GD. Biomechanical factors in youth sports injuries: a review. *Am J Sports Med*. (2021) 49:3456–65. doi: 10.1177/03635465211030567”. It should be “Hewett TE, Myer GD, Ford KR. Anterior cruciate ligament injuries in female athletes: part 1, mechanisms and risk factors. *Am J Sports Med*. (2006) 34:299–311. doi: 10.1177/0363546505284183”.

The reference for citation 33 was erroneously written as “Myer GD, Sugimoto D, Stracciolini A. Hormonal influences on injury risk in female adolescent athletes: a contemporary review. *J Pediatr Orthop*. (2023) 43:289–96. doi: 10.1097/BPO.0000000000002389”. It should be “Hewett TE, Myer GD, Ford KR. Decrease in neuromuscular control about the knee with maturation in female athletes. *J Bone Joint Surg Am*. (2004) 86:1601–8. doi: 10.2106/00004623-200408000-00001”.

The reference for citation 34 was erroneously written as “Read PJ, Oliver JL, Myer GD. Neuromuscular training and injury prevention in youth athletes. *Sports Med*. (2022) 52:611–23. doi: 10.1007/s40279-021-01587-8”. It should be “Hewett TE, Lindenfeld TN, Riccobene JV, Noyes FR. The effect of neuromuscular training on the incidence of knee injury in female athletes. A prospective study. *Am J Sports Med*. (1999) 27:699–706. doi: 10.1177/03635465990270060301”.

The reference for citation 35 was erroneously written as “Plisky PJ, Rauh MJ, Cameron KL. SEBT asymmetry and injury risk: updated evidence. *J Sport Rehabil*. (2020) 29:489–96. doi: 10.1123/jsr.2019-0123”. It should be “Stiffler MR, Bell DR, Sanfilippo JL, Hetzel SJ, Pickett KA, Heiderscheit BC. Star excursion balance test anterior asymmetry is associated with injury status in division I collegiate athletes. *J Orthop Sports Phys Ther*. (2017) 47:339–46. doi: 10.2519/jospt.2017.6974”.

The reference for citation 37 was erroneously written as “Gribble PA, Hertel J, Plisky P. Advances in dynamic balance assessment for injury prevention. *J Orthop Sports Phys Ther*. (2019) 49:401–9. doi: 10.2519/jospt.2019.0502”. It should be “Fullam K, Caulfield B, Coughlan GF, Delahunt E. Kinematic analysis of selected reach directions of the star excursion balance test compared with the Y-balance test. *J Sport Rehabil*. (2014) 23:27–35. doi: 10.1123/jsr.2012-0114”.

The reference for citation 38 was erroneously written as “Markovic G, Sarabon N, Mikulic P. Explosive power and injury risk in adolescent athletes. *Int J Sports Physiol Perform*. (2021) 16:523–30. doi: 10.1123/ijspp.2020-0456”. It should be “Bishop C, Turner A, Read P. Effects of inter-limb asymmetries on physical and sports performance: a systematic review. *J Sports Sci*. (2018) 36:1135–44. doi: 10.1080/02640414.2017.1361894”.

The reference for citation 40 was erroneously written as “Caswell SV, Cortes N, Chinn L. Predictive validity of functional movement screening in youth athletes. *J Athl Train*. (2019) 54:876–82. doi: 10.4085/1062-6050-44-18”. It should be “Kiesel KB, Butler RJ, Plisky PJ. Prediction of injury by limited and asymmetrical fundamental movement patterns in American football players. *J Sport Rehabil*. (2014) 23:88–94. doi: 10.1123/jsr.2012-0130”.

The reference for citation 41 was erroneously written as “Myer GD, Faigenbaum AD, Edwards NM. Neuromuscular training for injury prevention in youth sports. *Pediatrics*. (2020) 145:e20193542. doi: 10.1542/peds.2019-3542”. It should be “Emery CA, Roy TO, Whittaker JL, Nettel-Aguirre A, van Mechelen W. Neuromuscular training injury prevention strategies in youth sport: a systematic review and meta-analysis. *Br J Sports Med*. (2015) 49:865–70. doi: 10.1136/bjsports-2015-094639”.

The reference for citation 42 was erroneously written as “Velarde-Sotres A, Bores-Calle A, Mayordomo R. Jump performance and injury risk in adolescent volleyball players. *J Hum Kinet*. (2023) 87:123–32. doi: 10.5114/jhk/162456”. It should be “de Azevedo Sodré Silva A, Sassi LB, Martins TB, de Menezes FS, Migliorini F, Maffulli N, et al. Epidemiology of injuries in young volleyball athletes: a systematic review. *J Orthop Surg Res*. (2023) 18:748. doi: 10.1186/s13018-023-04224-3”.

The reference for citation 43 was erroneously written as “World Health Organization. *Global action plan on physical activity 2018–2030: More active people for a healthier world*. Geneva: WHO (2023).” It should be “World Health Organization. *Global Action Plan on Physical Activity 2018–2030: More Active People for a Healthier World*. Geneva: World Health Organization (2019).”

The reference for citation 44 was erroneously written as “Jandhyala A, Elahi J, Ganti L, McAuley D. Volleyball related injuries in adolescents: a decade of data. *Orthop Rev*. (2024) 23:123665. doi: 10.52965/001c.123665”. It should be “Jandhyala A, Elahi J, Ganti L, McAuley D. Volleyball related injuries in adolescents: a decade of data. *Orthop Rev*. (2024) 16:123665. doi: 10.52965/001c.123665”.

The **Generative AI statement** was erroneously given as “The authors declare that no Gen AI was used in the creation of this manuscript.” The correct **Generative AI statement** is “The author(s) declared that generative AI was used in the creation of this manuscript. A generative AI tool was used to format the manuscript and its reference list according to the journal's style requirements. During the preparation of the correction notice to this article (10.3389/fpubh.2026.2007172), a generative AI tool (Claude, Anthropic) was also used to assist in checking the bibliographic details of the reference list against the original sources; all corrected references were reviewed by the authors. The scientific content, data analysis and interpretation are entirely the work of the authors, who take full responsibility for the content of this article.”

The original version of this article has been updated.

